# Modulation of Lithium Disilicate Translucency through Heat Treatment

**DOI:** 10.3390/ma14092094

**Published:** 2021-04-21

**Authors:** Seok-Ki Jung, Dae Woon Kim, Jeongyol Lee, Selvaponpriya Ramasamy, Hyun Sik Kim, Jae Jun Ryu, Ji Suk Shim

**Affiliations:** 1 Department of Orthodontics, Korea University Guro Hospital, Seoul 08308, Korea; jgosggg@korea.ac.kr; 2 Department of Medicine, Korea University Graduate School, Seoul 02841, Korea; damdeok28@daum.net (D.W.K.); dr.selvaponpriya@gmail.com (S.R.); 3 Department of Prosthodontics, Korea University Guro Hospital, Seoul 08308, Korea; wddc@korea.ac.kr; 4 Korea Institute of Ceramic Engineering and Technology, Jinju-si 52851, Korea; hyunkim@kicet.re.kr; 5 Department of Prosthodontics, Korea University Anam Hospital, Seoul 02841, Korea

**Keywords:** glass ceramics, grain size, optical properties, strength

## Abstract

The aim of this study was to present a control method for modulating the translucency of lithium disilicate ceramics through thermal refinement. Identical lithium disilicate blocks were thermally refined using four different heat treatment schedules, and the microstructure, translucency, and flexural strength of the ceramics were investigated in detail by SEM, spectroscopy, and a piston-on-three-ball test. The results showed that ceramics treated under higher heat had larger grains, with an average size between 240 and 1080 nm. In addition, a higher transmittance of all wavelengths was observed in ceramics treated under lower heat, and the transmittance in the 550 nm wavelength ranged from 27 to 34%. The results suggest that the translucency of ceramics can be modified through thermal refinement under two conditions: (1) the particle size of the ceramic is small enough to achieve minimal grain-boundary light scattering, and (2) the percentage of particles allowing visible light transmission is altered by the heat treatment.

## 1. Introduction

Lithium disilicate, which has a major crystalline phase of Li_2_Si_2_O_5_ [1], shows a typical microstructure where elongated crystals form an interlocking pattern [2,3]. Since lithium disilicate was first introduced as a dental restorative material by Ivoclar Vivadent, the material has been popular due to its favorable optical characteristics and high mechanical strength [4]. “Ingot-type” lithium disilicate has been used in the conventional dental fixed-restoration fabricating method known as the lost wax technique. Recently, with the advance of CAD/CAM (computer-aided design and computer-aided manufacturing) technology, a lithium disilicate block (IPS™ e.Max CAD) has been introduced for use in milling procedures. For easy milling, increased cutting efficiency, and minimal waste of milling tools, the lithium disilicate block is used in the intermediate state of crystallization (Li_2_SiO_3_) [5], and additional thermal refinement processes are necessary after milling to enrich their crystallization [5].

Microstructure plays a major role in determining the translucency of ceramics, and this translucency can be modified by varying the volume, size, and density of crystals [6]. A fine-grained microstructure is desirable in order to improve the translucency in glass ceramics [6]. Ceramics with crystallites of a dimension smaller than the wavelength of light especially show improved translucency [7]. A microstructure with a high crystal density makes the ceramic less translucent as the light scattering is decreased [8,9]. Although the translucency of a ceramic can also be modified by adding pigments into the glass frit, the final results are more dependent on the phase composition and microstructure of the glass than on influences from a specific compound [5].

The microstructure of ceramics can be modified by adding chemical components or controlling the heat treatment [10,11]. Nucleating agents used for tailoring ceramic crystallization control both nucleation and crystal growth processes and determine the final shape, size, and contents of the crystal [12]; for example, CaO, P_2_O_5_, and CaF_2_ all induce internal nucleation, and affect the morphology of crystals in lithium disilicate after heat treatment [13,14,15]. In high concentrations, nucleating agents cause the microstructure of lithium disilicate to become denser, and the crystals smaller and more spherically shaped [16]. On the other hand, ZrO_2_ is used to control phase composition by altering the crystallization kinetics that reduce the content of lithium metasilicate and hamper the growth of lithium disilicate [11]. Heat treatment is used to dissolve lithium metasilicate and to crystalize lithium disilicate [1]. Different heating parameters can change the driving force for dissolving lithium metasilicate and altering the overall phase composition [17,18]. Temperature is highly influential to glass viscosity and the mobility of molecules in ceramics; a high-temperature treatment causes low glass viscosity and high mobility of molecules in a ceramic and induces a coarsening process that facilitates crystal growth [19]. Holding time is related to the total energy applied to ceramics and increasing holding time during the nucleation stage causes an increased number of crystallites [19]. A controlled coarsening process during nucleation is achieved with an optimum nucleation duration to produce a fine-grained glass ceramic [20]. The heating schedule can be divided into a one- or two-stage process: the initial heat treatment critically acts to establish a setting for stabilizing lithium metasilicates, and the second heat treatment supplies the thermal energy to destabilize lithium metasilicate and induce the growth of lithium disilicate [1]. Previous studies have demonstrated that a two-stage heating schedule results in the formation of more and larger crystals than a single-stage heating schedule [1,17,18,21].

In the present study, we used a controlled thermal refinement process to modulate translucency in lithium disilicate ceramics with a submicron-microstructure and evaluated the reliability of the method. The null hypothesis was that the translucency of lithium disilicate would not be modified by a controlled thermal refinement. In addition, we evaluated the effect of the altered substructure of lithium disilicate by thermal refinement on its translucency and mechanical properties.

## 2. Materials and Methods

### 2.1. Specimen Preparation

#### 2.1.1. Ceramic Block Preparation

The glass batch (72.6% SiO_2_, 10.7% Al_2_O_3_, 7.9% K_2_O, 2.1% CaO, 0.3% TiO_2_, 4.7% Na_2_O, 1.1%, Li_2_O, and 0.5% MgO; mol%) was contained in a 100% Pt crucible (LT Metal, Seoul, Korea) and melted in an electric kiln (Fredrickson Kiln Co., Alfred, NY, USA) at 1316 °C for 7 h. Glass blocks fabricated from the batch were quenched in an air with the crucible and heat-treated for nucleation (Prototype, HASS Co., Gangneung, Korea).

#### 2.1.2. Heating Schedule

Table 1 shows the summary of the heating schedule. The prepared glass blocks were labeled 815T, 825T, 840T, and 860T according to the four different firing temperatures (815 °C, 825 °C, 840 °C, and 860 °C, respectively). They were then thermally refined in a furnace (Ivoclar Vivadent Programat, Ivoclar Vivadent AG, Schaan, Liechtenstein) as follows: after an initial standby duration of 3 min in 400 °C, the temperature was increased to each firing temperature at a rate of 60 °C/min. Holding time at each firing temperature was 15 min, and the open temperature of the furnace head was 690 °C.

### 2.2. Observation of Microstructure

Five discs for each group were used for the observation of microstructure. The glass-ceramic specimens were mechanically polished using a 1 μm diamond suspension and 4000-grit SiC paper, and etched using 40% hydrofluoric acid. Each specimen was then coated with platinum in an ion coater (Eiko IB-5, Tokyo, Japan) for observation by scanning electron microscopy (SEM; Hitachi S-4500, Tokyo, Japan). Micrographs (20,000×, area = 30.1 μm^2^) were used to quantify the crystal number; average, minimum, and maximum crystal size; percentage of typical-sized particles using a digital image analyzing software package (ImageJ, NIH, Bethesda, MD, USA). Samples of glass-ceramics were chosen randomly from blocks before and after heat-treated at each temperature and pulverized for X-ray diffraction analysis (XRD). The specimens were placed in the holder of a Multi Flex X-ray diffractometer (Rigaku, Tokyo, Japan) using flat plate geometry. Cu Kα radiation was used in the 2θ range of 10°–60°, with a scanning rate of 2°/min.

### 2.3. Measurement of Optical Characteristics and Mechanical Properties

#### 2.3.1. Visible Light Transmittance

The ceramic discs were specially designed with set dimensions of 10 mm diameter by 1 mm thickness to best measure translucency. To evaluate the consistency of the translucency of the ceramics, multiple discs were prepared through separate thermal refinement processes. In total, 24 discs were prepared; six discs were allocated for each translucency. Since translucency is an optical property, it was evaluated using a spectrophotometer (UV-2600; Shimadzu, Kyoto, Japan) using the transmittance mode at wavelengths of between 400 and 700 nm.

#### 2.3.2. Biaxial Flexural Strength

A piston-on-three-ball test (according to ISO 6872) [22] was performed to measure biaxial flexural strength. Ten ceramic discs for each group were prepared, with each disc having dimensions of 14 mm diameter by 1 mm thickness after preparation that included heat treatment, finishing, and polishing. The discs were positioned on a universal testing machine (AG-10kNX, Shimadzu Co, Japan) supported by three steel balls (3 mm diameter) positioned 120° apart on a support circle (12 mm diameter). A flat punch-shaped rod (1.2 mm in diameter) with a crosshead speed of 1 mm/min was used to place a load on the center of the specimens until a fracture occurred. The biaxial flexural strength in MPa was calculated from the peak load of failure.

### 2.4. Statistical Analysis

All data is shown as mean and standard deviation. To evaluate the statistical significance between the study groups, the Kruskal–Wallis test and post-hoc analysis using the Dwass–Steel–Critchlow–Fligner method were conducted. A *p*-value less than 0.05 was considered statistically significant. Statistical analyses were performed with SAS software (version 9.4; SAS Institute, Cary, NC, USA) [23]. To monitor the reliability of controlling ceramic translucency by thermal refinement, translucency results from six different specimens were evaluated using the intraclass correlation coefficient method with MedCalc statistical software (version 18; Ostend, Belgium) [24]. For assessing reliability between six specimens, the transmittance of each disc was repeatedly measured five times. The assessment of reproducibility of ceramic translucency by thermal refinement was evaluated through comparing the subjects from repeat measuring of six discs.

## 3. Results

### 3.1. SEM Analysis

**Figure 1:**
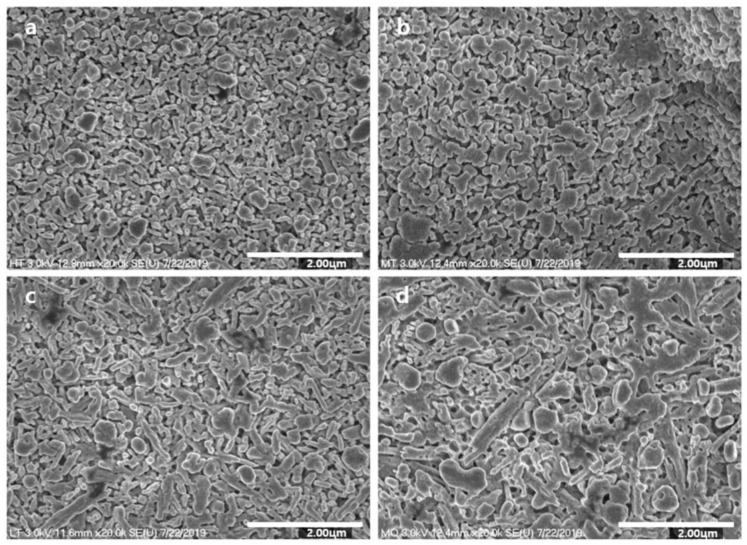
SEM photographs of lithium disilicate glass ceramics: (**a**) 815T, (**b**) 825T, (**c**), 840T, and (**d**) 860T. Original magnification 20,000×.

Figure 1 presents the observed microstructures of the specimens. While 815T exhibited a typical substructure consisting of fine, rounded particles, 825T consisted of particles larger than those of 815T. Both 815T and 825T showed relatively uniform patterning of their particle arrays, while 840T and 860T showed irregular patterning of spherical and elongated particles. 860T exhibited more irregular patterning and contained larger and more elongated particles than 840T.

Table 2 and Table 3 summarize the crystallization data and particle size ratios for the microstructure analysis. The list of the specimens in the order of highest to lowest number of small particles is: 815T, 825T, 840T, and 860T; with the average size being 240, 500, 740, and 1080 nm, respectively. All particles of 815T were smaller than 400 nm in diameter, and those of 825T were between 200 and 600 nm. Nearly all particles (90.91%) of 840T were between 200 and 800 nm, while the remaining particles (9.09%) were larger than 800 nm. More than half of the particles (58.82%) of 860T were between 200 and 800 nm, and the remaining particles (41.17%) were greater than 800 nm.

### 3.2. XRD Analysis

**Figure 2:**
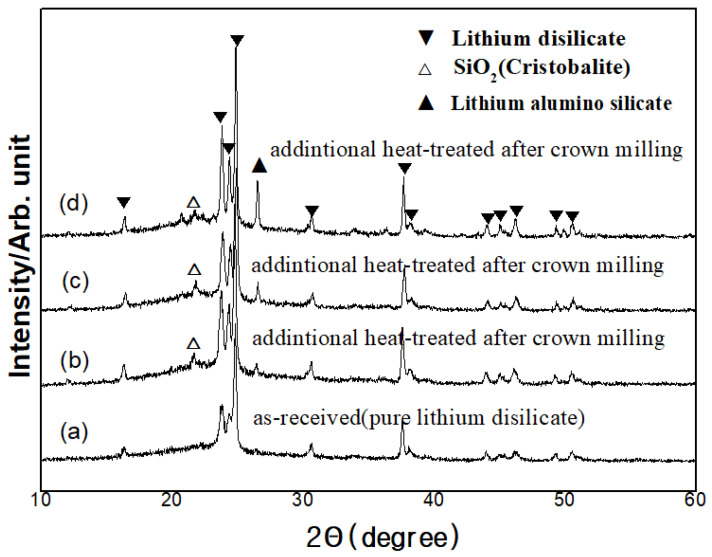
XRD of lithium disilicate glass ceramics: (**a**) glass-ceramic, (**b**) 815T, (**c**), 840T, and (**d**) 860T.

A characteristic XRD pattern after each heat treatment is presented in Figure 2. The main crystalline phase was lithium disilicate, and the major peaks of the phase were observed at the 2θ values of 23.781, 24.341 and 24.841. Blocks before heat treatment also showed the peak of lithium disilicate. Blocks treated with higher heat treatment showed higher peak of lithium disilicate. Cristobalite and lithium alumino silicate were shown as the secondary phases.

### 3.3. Translucency

**Figure 3:**
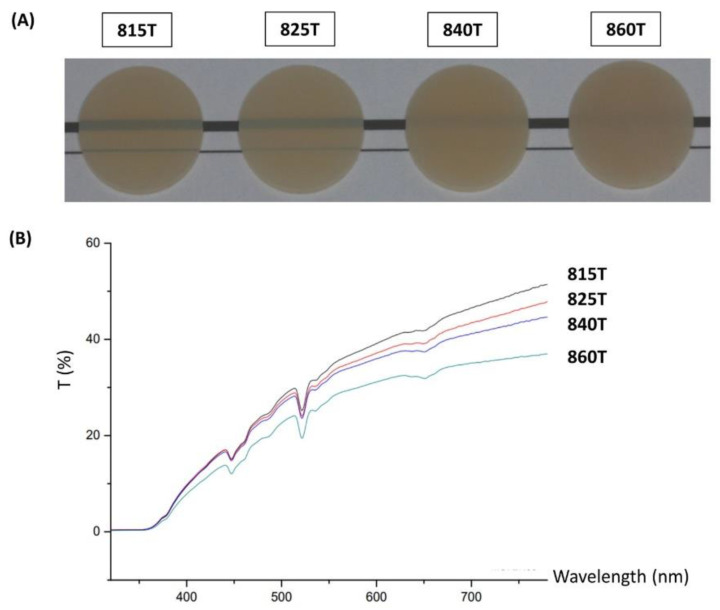
(**A**) Apparent translucency of ceramics. (**B**) Spectra of total light transmittance: the transmittance increased with the increase in light wavelength except at 450 and 530 nm. 815T was the most translucent group followed by 825T, 840T, and 860T.

Figure 3A shows the apparent translucency of each specimen and Figure 3B shows the spectra of light transmittance. Table 4 displays the light transmittance percentage along with the reliability of controlling the translucency of each ceramic. The highest transmittance of all wavelengths was observed in 815T, followed by 825T, 840T, and 860T. In the 550 nm wavelength, the transmittance of 815T, 825T, 840T, and 860T were 34.03, 32.65, 31.82, and 27.42, respectively, while the intraclass correlation coefficients were 0.95, 0.98, 0.97, and 0.97, respectively.

### 3.4. Biaxial Flexural Strength

**Figure 4:**
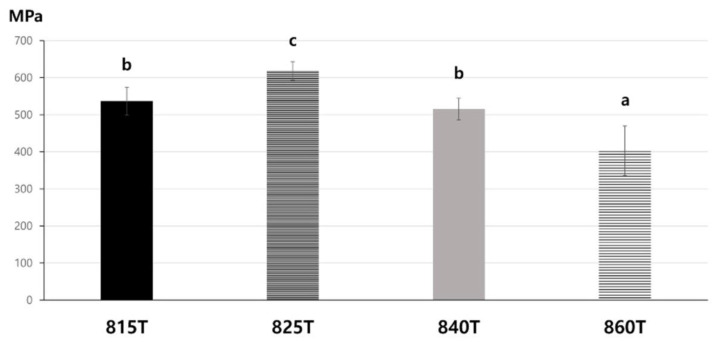
Flexural strength of the specimens in each group. Distinct letters indicate significant differences between groups. Groups with the same letter are not statistically different (*p* > 0.05). (**a**–**d**) The small caps indicate the statistically relation between sites. The same letters indicate non-significant difference between sites (*p* > 0.05)

Figure 4 presents the flexural strength of each group. In the piston-on-three-ball test, failure was considered to be reached when the specimens were fully broken. The highest flexural strength (617.88 MPa) was observed in 825T, and the lowest flexural strength (403.19 MPa) in 860T, both with statistical significance. There was no statistical difference between 815T (536.72 MPa) and 840T (515.13 MPa) (*p* > 0.05).

## 4. Discussion

Controlling translucency through the thermal refinement process is beneficial to CAD/CAM of lithium disilicates because one single block can be used for various clinical situations which require different levels of ceramic translucency. This is convenient since ceramics have to undergo thermal refinement during the CAD/CAM process. In this study, we evaluated the possibility of controlling translucency through thermal refinement. Through controlling heating parameters, ceramics with different translucencies with statistical significance were obtained. Therefore, the null hypothesis was rejected. In addition, high reproducibility in realizing each different translucency was shown (intraclass correlation coefficient values were above 0.95). Our results demonstrate that our method to modify the translucency of lithium disilicate through thermal refinement is efficient and reliable.

In addition, this study reliably demonstrates how translucency and flexural strength are modified by the alteration of the ceramic microstructure. The four different ceramics were materialized from blocks having identical components, and we altered the ceramic microstructures using heat treatment only. The ceramic blocks had completed nucleation through their initial heat treatment, and the additional thermal refinement modified the microstructure of the ceramics by affecting the process of crystallization according to the different temperatures. As noted in previous studies, a high temperature results in high mobility of molecules, low glass viscosity, and enhanced dislocation, thereby forming small crystal aggregates [19,25]. Thus, coarsening of the microstructure was observed on increasing the heat treatment temperature [26], and our findings were in agreement with this as the ceramics which were thermally refined at higher temperatures were composed of larger-sized particles.

Regarding translucency, many previous studies have shown modified translucency with supplemental materials such as CaO, P_2_O_5_, CaF_2_, and ZrO_2_. In this study, for the first time, the translucency of ceramics was significantly modified with high reproducibility only by altering their microstructures. When light moves from air into a solid, it is reflected, absorbed, or transmitted. The light transmitted to a ceramic may experience the typical processes of reflection and refraction, termed “scattering” [27]. The light scattering is determined by the characteristics of ceramics including their impurities, pores, defects, and grain boundaries [27]. Relying on this diffuse-transmission mechanism, increasing the grain size is favorable to encountering a less-powerful light beam at the grain boundaries, thus causing minimal grain-boundary light scattering [28]. Another method to achieve low grain-boundary light scattering is by maintaining a smaller grain size relative to the wavelength of light [29]. The Rayleigh–Gans–Debye scattering model, a theoretical model to predict the desirable grain size of ceramics for obtaining high translucency, shows that a smaller particle size relative to the wavelength of incident light is more likely to cause elastic scattering [29]. From the Rayleigh approximation, 2πr n/λ, where r is the radius of the grain, n is the refractive index, and λ is the wavelength of light [27], considering that the average refractive index of lithium disilicate is 1.55 in the visible light spectrum [30], the maximum grain size required for Rayleigh scattering to transmit for visible light (wavelength between 370 and 750 nm) is 483.87 nm. In this study, 815T, 825T, 840T, and 860T had grain sizes in the Rayleigh scattering ranges for visible light of 100%, 45%, 17%, and 8%, respectively. Overall, in spite of the small alteration in the size of ceramic particles through the coarsening process, there were definite differences between the compositions of grain sizes which critically affect the transmission of visible light.

In general, the mechanical properties of ceramic are affected by the crystal size [31], crystalline contents [32], and the irregularity of particles [33]. The ceramic composed of smaller particles shows better mechanical properties because the critical flaw size is proportional to the crystal size [34,35]. An increase in the crystalline content leads to improved mechanical properties of ceramics [36]. The ceramic composed with the particles of various size shows lower mechanical properties because irregular particle size induces stress, raising flaws, and breaking the interfacial interaction between the matrix and particles [33,37]. In this study, higher flexural strength was observed in the order of 825T, 815T/840T, and 860T, and the mechanical properties were determined by the three factors. 825T showed the highest flexural strength which was composed of uniformly small particles with high crystallinity. While 815T was composed of the uniformly small particles, it showed lower percentage of crystallinity than other groups. While 840T showed higher percentage of crystallinity, it was composed of irregular large particles. 860T comprised the most irregular and largest particles among the groups, and showed the lowest flexural strength.

The products of the method can be replicated for the commercial use of the control of translucency through thermal refinement. In this study, high reliability was obtained as shown by the intraclass correlation coefficient values of above 0.95. The high repeatability may be due to the consistent content and crystal size during the materialization of lithium disilicate [38]. In addition, crystal growth is more stable through the two-stage heat treatment. However, a limitation of this study is that only a single furnace was used for thermal refinement; there may be subtle differences between different furnaces which may affect results. Future research using various kinds of furnaces may be beneficial to verify these results. In addition, in order to obtain more representative results, a greater number of measurements should be carried out in the future study. Energy Dispersive X-ray Spectroscopy (EDS) was not carried out in this study. It might help to understand more clearly the cause of irregular strength behavior in the studied samples. Future research requires to consider these areas.

## 5. Conclusions

In this study, we verified a control method to modulate the translucency of lithium disilicate ceramics through thermal refinement processing after the milling process for a CAD/CAM of a ceramic block. The results of this study suggest that controlling the translucency of a ceramic through thermal refinement is possible under two conditions that (1) the particle size of the ceramic is small enough to achieve minimal grain-boundary light scattering, and (2) the percentage of particles allowing visible light transmission is altered by the heat treatment.

## Figures and Tables

**Table 1:** Heat treatment schedule.

Groups	Standby Temperature (°C)	Standby Duration (min)	Firing Temperature (°C/min)	Holding Time (min)	Vacuum on/off Temperature (°C)	Furnace Head Open Temperature (°C)
815T	400	3	815	15	550/815	690
825T	400	3	825	15	550/825	690
840T	400	3	840	15	550/840	690
860T	400	3	860	15	550/860	690

**Table 2:** Crystallization data for the glass ceramics.

Groups	Crystal Number Mean (SD)	Crystal Size (nm)				Distance between Crystals (nm)
		Mean	Min	Max	SD	
815T	2237.6 (89.2)	240	10	370	56.43	0.19 ± 0.05
825T	837.4 (56.1)	600	240	570	63.18	0.29 ± 0.06
840T	756.1 (58.6)	740	240	1450	167.25	0.34 ± 0.09
860T	330 (14.2)	1080	260	2760	584.36	0.43 ± 0.14

SD; standard deviation.

**Table 3:** Ratio of typical particle size (nm) per unit area.

Groups	<200	200–400	400–600	600–800	>800
815T	71.88%	28.12%	-	-	-
825T	-	42.42%	57.58%	-	-
840T	-	15.15%	51.52%	24.24%	9.09%
860T	-	5.88%	17.65%	35.29%	41.18%

**Table 4:** Light transmittance percentage and reliability of controlling translucency of each ceramic.

Results	815T	825T	840T	860T
Light transmittance percentage (%) *	30.50 ± 0.21 ^d^	27.58 ± 0.38 ^c^	26.28 ± 0.31 ^b^	22.20 ± 0.38 ^a^
Intraclass correlation coefficient ^†^	0.95	0.98	0.97	0.97

* Groups with the same letter are not statistically different (*p* > 0.05). ^†^ The value of 1 means the highest reliability of translucency, ^a,b,c,d^ The small caps indicates the statistically relation between groups. The same letters indicate non-significant difference between sites (*p* > 0.05).

## Data Availability

The data underlying this article will be shared on reasonable request from the corresponding author.
